# Neuromuscular adaptation and postural muscle activity patterns during visual virtual reality stimulation: age-related differences between young and older adults

**DOI:** 10.1007/s00221-026-07263-4

**Published:** 2026-03-17

**Authors:** Eva Ekvall Hansson, Jenny Älmqvist Nae, Elin Östlind, Ulrik Röijezon, Per-Anders Fransson

**Affiliations:** 1 Department of Health Sciences, Lund University, Lund, Sweden; 2 Department of Otorhinolaryngology Head and Neck Surgery, Skåne University Hospital, Lund, Sweden; 3 Department of Clinical Sciences Lund, Lund University, Lund, Sweden; 4 Department of Health, Education and Technology, Luleå University of Technology, Luleå, Sweden

**Keywords:** Ageing, Muscle activity patterns, Postural stability, Virtual reality, Vision

## Abstract

Vision provides important sensory input to postural control. Medical conditions and aging affect sensory—Central Nervous System (CNS) interactions, causing mismatches. Virtual Reality (VR) may address these by promoting recalibration from vision to other systems. Twenty-eight young adults (17 males, mean age 25.3 years) and 25 older adults (14 males, mean age 74.8 years) were included. Electromyography (EMG) was recorded while the participants performed, while standing, 2 control tests in quiet stance with eyes open and closed and thereafter repeatedly five times watching the same 120-second VR simulation of a rollercoaster ride. The older group watched a less challenging rollercoaster ride. Muscle activation patterns in four postural muscles; gluteus medius, medial gastrocnemius, tibialis anterior, and peroneus longus. were recorded bilaterally. The first VR session produced a marked stability challenge, reflected by significantly increased mean (*p* ≤ 0.008) and SD (*p* < 0.001) EMG amplitudes in both young and older adults in all four muscles. Both mean (*p* < 0.001) and SD (*p* < 0.001) EMG amplitudes increased more among the older adults than in young adults in all four muscles. Repeated VR sessions led to progressively decreased mean (*p* < 0.001) and SD (*p* ≤ 0.003) EMG amplitudes compare to the first VR session in both young and older adults in all muscle groups. Both age groups could quickly reduce the effects of the VR challenges when allowed to familiarize themselves with the situation during repeated sessions. Postural control adaptations enabled modulation of both the degree of muscle activation measured with EMG amplitudes and muscle group activity patterns to address stability challenges.

## Introduction

Postural balance is maintained through the complex integration of visual, vestibular, and somatosensory inputs, central nervous system (CNS) processing, weighting, and well adapted motor commands to postural muscles (Horak 2006). Any discrepancy or conflict among the senses can destabilize standing balance as the CNS attempts to recalibrate sensory weights (Bugnariu and Fung 2007). Visual information plays a crucial role in postural control—classic experiments (e.g. the “moving room” paradigm) demonstrated that simply perceiving a moving visual scene can induce a strong illusion of self-motion and cause compensatory postural instability (Schoner 1991). In everyday life, a familiar example is the vertiginous sensation of self-motion experienced when a neighboring train starts moving; the moving visual field can trick the brain and momentarily disrupt balance. Under normal conditions, healthy adults can usually resolve such sensory conflicts quickly, but the degree of imbalance and the adaptation process can vary with context and with individual factors such as age (Bugnariu and Fung 2007).

Virtual reality (VR) technology provides a powerful tool to simulate immersive visual environments and induce controlled sensory conflicts without physical movement. By presenting visual motion through a head-mounted display, VR can evoke postural responses analogous to real-world perturbations (Bugnariu and Fung 2007; Fransson et al. 2019). This makes VR an attractive experimental paradigm for studying how the CNS reweights sensory inputs to maintain balance. Notably, prolonged or repeated exposure to the same provocative visual stimulus can engage adaptive processes (Bugnariu and Fung 2007; Fransson et al. 2019). The CNS may learn to down-weight the misleading visual cues and rely more on somatosensory and vestibular information, thereby gradually reducing postural instability. Several studies have observed that healthy young adults (Fransson et al. 2019) as well as older adults (Almqvist Nae et al. 2024) show a habituation effect with repeated VR perturbations, but exceptions to this notion also exist (Ahuja et al. 2025). For example, Fransson et al. exposed participants to a rollercoaster VR scene and found an immediate increase in energy used towards the support surface for maintaining postural stability, followed by significant reduction (47–62% decrease in energy) after 4–5 repeated exposures (Fransson et al. 2019). Such findings suggest that humans can adapt relatively quickly to recurrent visual stimulation, a process consistent with sensory reweighting theories.

However, while the effects of VR-induced sensory conflicts on postural stability or sway are documented, less is known about the corresponding neuromuscular responses—i.e. how the activation of key postural muscles is modulated during these visual stimulations and through adaptation. A previous study showed differences in postural muscle onset between younger and older adults due to visual stimulation and surface perturbations (Bugnariu and Fung 2007). However, the change in EMG amplitude due to VR-induced visual stimulation in standing balance test has not, to our knowledge been evaluated, in young and older healthy adults. Aging is associated with a decline in sensory functions and slower reflexes, which often lead older adults to rely more on visual cues for balance. When visual information is removed or distorted, older individuals typically show disproportionately larger instability or sway compared to young adults. Bugnariu and Fung (2007) demonstrated that older adults relied more on vision than younger adults during standing and had greater difficulty resolving visual–vestibular conflicts; nevertheless, with repeated exposures even older participants were able to recalibrate their sensory weighting and improve their balance within a single extended VR session (Bugnariu and Fung 2007). These findings raise the question of how older people respond neuromuscularly to novel VR perturbations and whether they exhibit the same capacity for adaptation over repeated trials as younger people. On one hand, greater initial muscle activation might be expected in older adults as a compensatory strategy for instability, since advanced age is associated with increased muscle co-activation and stiffness during postural tasks (Nagai et al. 2011). On the other hand, the plasticity for adaptation might be reduced or slower in older adults due to age-related declines in sensory reweighting efficiency (Rubega et al. 2021). Understanding these age differences is not only scientifically important but also relevant for applying VR in balance rehabilitation of older people.

The objective of the present study was to increase knowledge on visual VR stimulation effects on postural muscle activity in standing, compared to normal quiet stance, and whether repeated exposures to the same VR stimulus would lead to adaptive changes in muscle activation among young and older adults. We hypothesized that the introduction of an immersive VR stimulus (a 360° rollercoaster simulation) would acutely increase muscle activity, reflecting the effort to counteract the visually induced imbalance. Furthermore, we expected that repeated trials of the same VR stimulus would induce a habituation response, evidenced by progressively lower muscle activation, due to sensorimotor adaptations. Finally, we expected that older adults would present relatively larger EMG amplitude compared to young adults, due to age related declines in sensorimotor functions. This work is novel in its focus on neuromuscular adaptation to repeated VR perturbations and in directly comparing young vs. older adults in the same experimental paradigm. Ultimately, these findings may improve the design of VR-based balance training programs by elucidating how the body’s stabilizing muscles respond and adapt to challenging immersive visual environments.

## Materials and methods

### Ethical consideration

The study was performed in accordance with the latest Declaration of Helsinki and received ethical approval from the Ethical Review Authority in Sweden (Dnr:2023-00353-01). All participants gave their written consent prior to participation.

### Participants

Participants were included in the young group if they were between 18 and 45 years old and in the older group if they were above 65 years old. A general inclusion criterion for both young and older participants were that they were able to read and write in Swedish, considered themselves healthy and had no prior history of diseases that could affect their current balance, e.g., a known history of vertigo, musculoskeletal or neurological diseases. The participants were screened for health disorders by using a custom-made questionnaire, e.g., whether they had reduced balance, prior injury in the lower extremity the last year. When screened for prior experience of watching VR movies, 17 of 28 participants in the younger group and 9 of 25 in the older group had tested it one time, and a few participants in each group had used VR two times. The recruitment of participants was conducted through social media platform advertisements (Facebook) and through contacts with colleagues, friends and family. Those who accepted to participate in the study were referred to the Movement and Reality Laboratory (MoRe-lab) at Lund University for the testing.

Thirty young participants were recruited to participate in the study, whereof 28 were included in the final analysis (17 males, 11 females, mean age 25.3 years (standard deviation (SD) 4.6 years), mean height 1.80 m (SD 0.11 m), mean weight 75.8 kg (SD 13.5 kg), mean BMI 23.4 (SD 2.8)). Two young participants were excluded due to technical issues. Thirty-two older individuals were recruited to participate in the study, whereof 25 were included in the final analysis (14 males, 11 females, mean age 74.8 years (SD 3.5 years), mean height 1.72 m (SD 0.09 m), mean weight 73.4 kg (SD 11.3 kg), mean BMI 24.8 (SD 3.6)). Five older participants were excluded because they were unable to properly perform the tests, e.g., by taking steps during one or more assessments. Two older participants were excluded due to technical issues.

### EMG assessment

The study hardware was designed to sample data at 2000 Hz from an electromyography (EMG) system. The EMG system was a wireless Noraxon Ultium system with 16 channels (USA. Inc, Scottdale, Arizona, USA), set to lowpass filter at 500 Hz sampled muscle activity before analysis. The skin was shaved and abraded using a medical abrasion gel (Nuprep, Weaver and Company, Aurora, Colorado, USA) before electrode placement. Dual surface electrodes with an inter-electrode distance of 20 mm (Noraxon, USA, Inc) were placed bilaterally on the postural muscles gluteus medius (GlutMed), medial gastrocnemius (MedGas), tibialis anterior (TibAnt), and peroneus longus (PerLong), according to the SENIAM surface electromyography for non-invasive assessment of muscles guidelines (Hermens et al. 2000).

Prior to conducting the study assessments, the maximum voluntary contraction (MVC) was determined for each target muscle in accordance with the SENIAM guidelines (Hermens et al. 2000). Specifically, hip abduction was used to assess the MVC of the GlutMed, plantar flexion for the MedGas, dorsiflexion for the TibAnt, and foot pronation for the PerLong. The procedure followed the SENIAM guidelines (Hermens et al. 2000), with a modification for GlutMed MVC, which was performed in supine position. The MVC assessments were repeated three times for each muscle with 30 s rest between contractions. During each trial, participants were instructed to gradually increase force and perform the contraction with maximal voluntary effort.

### Test procedure

All preparatory procedures and test sessions were conducted on the same day, with the entire protocol requiring approximately 90 min per participant. Each participant completed seven postural stability assessments, each lasting 2 min. Participants performed all 7 tests without shoes, with foot positioning standardized to achieve a slight outward rotation of approximately 30 degrees, in accordance with established protocols (Patel et al. 2009a, 2020; Fransson et al. 2019). The participants were instructed to stand with their arms folded across the chest and to maintain their head in a neutral, forward-facing position throughout the assessments. During the VR sessions the operator continuously monitored the participant’s stability and position of the head, and thus, was prepared to immediately address any issues.

Initially, two control tests, quiet stance with eyes open and quiet stance with eyes closed, were performed in a randomized order prior to the five virtual reality (VR) sessions. During the control tests, participants were instructed to stand once with their eyes closed and once while focusing on a visual target placed at eye level, approximately 5 m in front of them on the wall. The control tests served as a baseline reference for muscle activation patterns, which were later compared to those recorded during exposure to VR-induced visual stimulations. VR equipment was not used during the control tests to ensure accurate visual feedback under natural conditions.

The VR assessments were conducted using the Oculus Quest 2 headset (Facebook Technologies, LLC, Menlo Park, California, USA), which provides full 360° head tracking. Any head movement corresponded directly to changes in the displayed VR environment. To minimize variability and potential discomfort associated with excessive head movements, participants were instructed to keep their gaze fixed straight ahead throughout the VR sessions.

Prior to the VR sessions, participants passively viewed a 360° movie to calibrate the image clarity of the VR headset for each participant. Five postural stability assessments were then performed while participants took part in a VR session consisting of a 2-minute 360° rollercoaster movie. The same movie was used for each participant across the five postural stability sessions. The movie included rapid lateral turns and vertical ascents and descents to challenge postural control. Both older and younger participants viewed similar, though not identical, VR movies. The older participants viewed a less challenging movie compared to the young adults, to reduce potential strain and inability to perform the task. Participants were permitted to sit and rest 10 min between VR sessions as needed.

### EMG analysis

Surface EMG signals were recorded from GlutMed, MedGas, TibAnt, and PerLong muscles. The raw EMG signals were initially processed using a band-pass filter with cutoff frequencies set at 20 Hz and 500 Hz. The upper root mean square (RMS) envelope of the EMG signal was then computed using a sliding window of 75 milliseconds to capture the muscle activity amplitudes.

MVC values for each muscle were determined from the MVC assessments. The MVC data was processed with 20–500 Hz band pass filter and RMS using a 75-millisecond sliding window. Thereafter we used a moving average filter with a 1-second sliding window, after which the peak value within the filtered signal was identified as the MVC for each muscle. The EMG data during the VR-tests were processed with the same bandpass filter and RMS procedure as described above. To normalize the EMG data, the amplitude of the EMG signals during each test were divided by the corresponding MVC value for each muscle. The values presented in the figures have been multiplied by 100, and thus, the normalized values are presented as %MVC. The mean and SD of the normalized EMG amplitudes were calculated for each leg. Subsequently, the mean values for each muscle across both legs were derived and used for further analysis.

### Statistical analysis

A repeated measures General Linear Model (GLM) Analysis of Variance (ANOVA) analysis was used for evaluating the performance across repeated VR sessions. Prior to using this analysis method, the normality or close to normality of the residuals from all model combinations was confirmed, ensuring the suitability of the GLM ANOVA analysis for the datasets. The primary dependent variables in this study were the log-transformed mean and SD of the normalized EMG amplitudes for the GlutMed, MedGas, TibAnt, and PerLong muscles.

For the VR sessions, the main factors included Repetition (Sessions 1–5; degrees of freedom [d.f.] = 4), Age Group (Young vs. Older; d.f. = 1), and their interaction (Repetition × Age Group). In this model, Repetition was treated as a within-subjects factor, and Age Group as a between-groups factor. For the control tests, the main factors analyzed were Vision (Eyes Open vs. Eyes Closed; d.f. = 1), Age Group (Young vs. Older; d.f. = 1), and their interaction (Vision × Age Group), with Vision treated as a within-subjects factor and Age Group as a between-groups factor.

Post hoc within-subjects analyses were conducted using the Wilcoxon matched-pairs signed-rank test (exact significance, two-tailed). These comparisons included:

- Changes between VR Session 1 and VR Session 5 to assess potential adaptation across repeated VR exposures.
- Differences between VR Session 1 and the Eyes Open control condition.
- Differences between VR Session 5 and the Eyes Open control condition.
- Comparisons between Eyes Open and Eyes Closed control conditions to evaluate the influence of vision (Patel et al. 2008).

Statistical significance for the GLM ANOVA was set at *p* < 0.05. For post hoc analyses, significance thresholds were adjusted using Bonferroni correction: *p* < 0.025 for VR-related comparisons and *p* < 0.05 for control test comparisons. Non-parametric tests were selected for post hoc analyses due to non-normal data distributions in some comparisons, as indicated by the Shapiro–Wilk test, which could not be resolved through log transformation (Altman 1991).

Sample size estimation based on posturography parameters indicated an effect size of 0.95, suggesting that with a significance level of *p* = 0.05 (two-tailed), a minimum of 11 participants would be required to achieve a statistical power of 0.80 for the primary outcome measures. Of note, the power calculation is indirect with respect to the primary EMG variables analyzed in the present study. All statistical analyses were performed using SPSS software (Version 28, IBM Corp., Armonk, NY, USA), and power analyses were conducted using G*Power (Version 3.1.9.7).

## Results

### Effects on EMG amplitudes from repeated VR sessions and from age

**Fig. 1:**
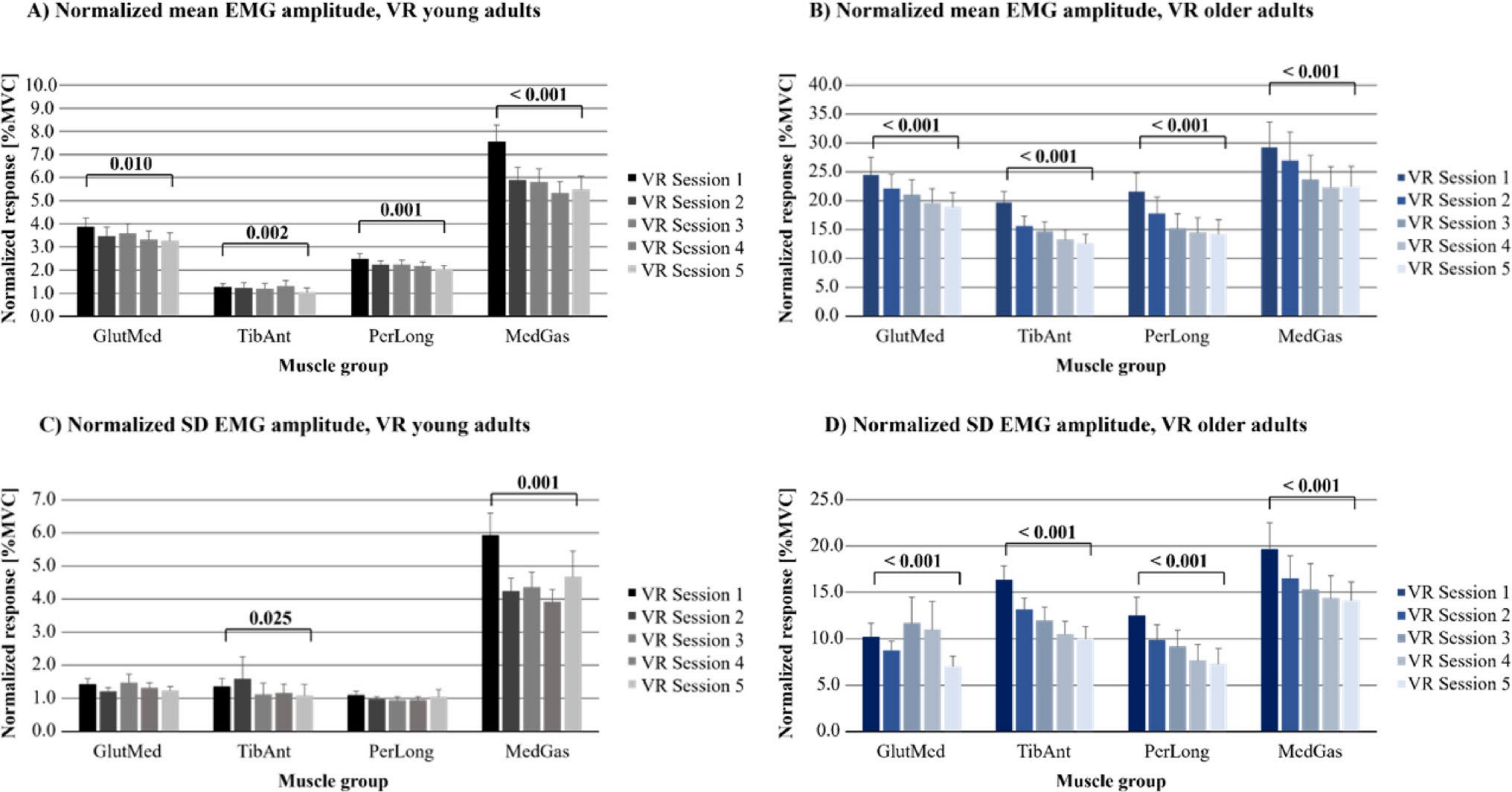
Normalized EMG amplitudes during repeated VR sessions in young and older adults. The values are presented on the scale of %MVC, as they are normalized with the corresponding muscle MVC. The results are presented as bars (mean) and whiskers (SEM) values. Values are presented for: **A** Normalized mean EMG amplitudes in young adults; **B** Normalized mean EMG amplitudes in older adults; **C** Normalized SD EMG amplitudes in young adults, and **D** Normalized SD EMG amplitudes in older adults

Analyses, using the repeated measures GLM ANOVA, revealed that when the participants repeatedly watched the same VR movie, the mean (*p* < 0.001) and SD (*p* ≤ 0.003) EMG amplitudes gradually decreased significantly from VR session 1 to VR session 5 in all investigated muscle groups (Fig. 1; Table 1). The mean (*p* < 0.001) and SD (*p* < 0.001) EMG amplitudes were significantly higher in the older group than in the younger group.

Significant interactions between repetition and age group revealed that repetition led to a significantly greater decrease from VR session 1 to VR session 5 in the older group than the younger group in mean EMG amplitudes in the TibAnt, PerLong, and MedGas muscles (*p* ≤ 0.036), as well as in the standard deviation of EMG amplitudes in the PerLong and MedGas (*p* ≤ 0.005), (Table 1).

**Table 1:** Effects on normalized EMG amplitudes from repeated VR sessions and from age

Normalized EMG amplitudes^a^	Mean^b^			SD^b^		
	Repetition	Age group	Repetition x Age group	Repetition	Age group	Repetition x Age group
GlutMed	**< 0.001 [28.1]**	**< 0.001 [126.9]**	0.136 [2.3]	**0.003 [9.5]**	**< 0.001 [129.0]**	0.113 [2.6]
TibAnt	**< 0.001 [21.8]**	**< 0.001 [253.6]**	**0.036 [4.6]**	**< 0.001 [14.4]**	**< 0.001 [195.0]**	0.132 [2.3]
PerLong	**< 0.001 [51.8]**	**< 0.001 [107.6]**	**< 0.001 [14.2]**	**< 0.001 [27.7]**	**< 0.001 [112.1]**	**0.004 [9.1]**
MedGas	**< 0.001 [34.8]**	**< 0.001 [49.3]**	**0.015 [6.4]**	**< 0.001 [25.1]**	**< 0.001 [40.4]**	**0.005 [8.7]**

^a^2-way repeated measures GLM ANOVA analyses of how the normalized EMG amplitudes was affected by main factor “Repetition”, ‘Age group’ and the interaction factor ‘Repetition’ x ‘Age group’ during VR

^b^Presented as: p-values and [F-values]. Found significant effects are marked in bold text

### Initial effects of VR and effects of adaptation to VR in young adults

**Fig. 2:**
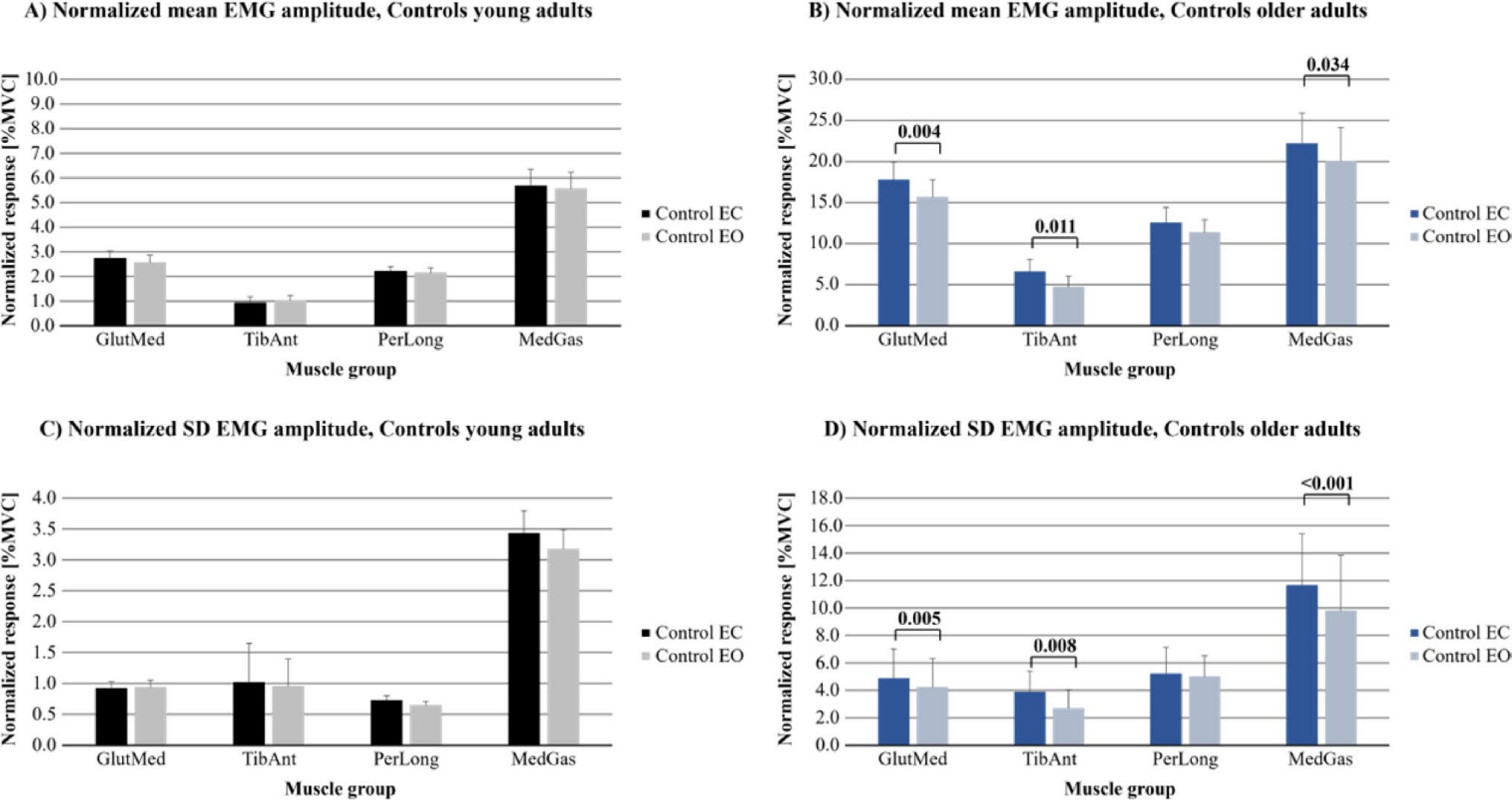
Normalized EMG amplitudes during the control tests in young and older adults. The values are presented on the scale of %MVC, as they are normalized with the corresponding muscle MVC. The results are presented as bars (mean) and whiskers (SEM) values. Values are presented for: **A** Normalized mean EMG amplitudes in young adults; **B** Normalized mean EMG amplitudes in older adults; **C** Normalized SD EMG amplitudes in young adults, and **D** Normalized SD EMG amplitudes in older adults

Within-subjects analyses revealed that the young participants used significantly higher mean (*p* ≤ 0.008) and SD (*p* < 0.001) normalized EMG amplitudes during VR session 1 compared to during the control test with eyes open in the four muscles investigated. Mean amplitudes were 51% higher in GlutMed; 23% higher in TibAnt; 15% higher in PerLong, and 36% higher in MedGas (Figs. 1 and 2; Table 2). SD EMG amplitudes were 52% higher in GlutMed; 41% higher in TibAnt; 69% higher in PerLong, and 87% higher in MedGas.

**Table 2:** Initial effects of VR and effects of adaptation to VR in young adults

Normalized EMG amplitude^a^		*p*-value	VR 1/EO^b^	*p*-value	VR 5/VR 1^c^	*p*-value	VR 5/EO^d^
Mean							
	GlutMed	**< 0.001**	**1.51 (0.11)**	**0.010**	**0.84 (0.06)**	**< 0.001**	**1.27 (0.11)**
	TibAnt	**0.002**	**1.23 (0.27)**	**0.002**	**0.82 (0.17)**	0.227	1.01 (0.11)
	PerLong	**0.008**	**1.15 (0.08)**	**0.001**	**0.82 (0.04)**	0.441	0.95 (0.06)
	MedGas	**< 0.001**	**1.36 (0.23)**	**< 0.001**	**0.73 (0.06)**	0.849	0.99 (0.14)
SD							
	GlutMed	**< 0.001**	**1.52 (0.21)**	0.227	0.87 (0.07)	**< 0.001**	**1.32 (0.10)**
	TibAnt	**< 0.001**	**1.41 (2.14)**	**0.025**	**0.80 (0.63)**	**0.025**	**1.14 (0.87)**
	PerLong	**< 0.001**	**1.69 (0.22)**	0.077	0.96 (0.18)	**0.010**	**1.61 (0.23)**
	MedGas	**< 0.001**	**1.87 (0.41)**	**0.001**	**0.79 (0.14)**	**< 0.001**	**1.47 (0.26)**

^a^Presented as quotient between the mean values for each dataset (SEM of individual quotients on sample level)

^b^Quotient between VR session 1/control test with eyes open

^c^Quotient between VR session 5/VR session 1

^d^Quotient between VR session 5/control test with eyes open. Statistically significant p-values are shown in bold.

The analyses to determine the effects of adaptation revealed that young participants significantly decreased normalized EMG amplitudes across the five VR sessions in the four muscles investigated (*p* ≤ 0.010) (Fig. 1; Table 2). Mean amplitudes were 16% gradually decreased from VR session 1 to VR session 5 in GlutMed; 18% decreased in TibAnt; 18% decreased in PerLong, and 27% decreased in MedGas. SD EMG amplitudes were 20% decreased in TibAnt and 21% decreased in MedGas (*p* ≤ 0.025).

When comparing the mean and SD EMG amplitudes during VR session 5 with the values during the control test with eyes open, mean EMG amplitudes remained significantly 27% higher in GlutMed (*p* < 0.001), but not in the other muscles (Figs. 1 and 2; Table 2). However, SD EMG amplitudes in the four investigated muscles remained significantly higher; 32% higher in GlutMed; 14% higher in TibAnt; 61% higher in PerLong, and 47% higher in MedGas (*p* ≤  0.025).

### Initial effects of VR and effects of adaptation to VR in older adults

Within-subjects analyses revealed that the older participants used significantly higher mean (*p* < 0.001) and SD (*p* < 0.001) normalized EMG amplitudes during VR session 1 compared to during the control test with eyes open in the four muscles investigated. Mean amplitudes were 56% higher in GlutMed; 320% higher in TibAnt; 90% higher in PerLong, and 45% higher in MedGas (Figs. 1 and 2; Table 3). SD EMG amplitudes were 141% higher in GlutMed; 510% higher in TibAnt; 151% higher in PerLong, and 100% higher in MedGas.

**Table 3:** Initial effects of VR and effects of adaptation to VR in older adults

Normalized EMG amplitude^a^		*p*-value	VR 1/EO^b^	*p*-value	VR 5/VR 1^c^	*p*-value	VR 5/EO^d^
Mean							
	GlutMed	**< 0.001**	**1.56 (0.21)**	**< 0.001**	**0.77 (0.03)**	**< 0.001**	**1.21 (0.16)**
	TibAnt	**< 0.001**	**4.20 (2.51)**	**< 0.001**	**0.64 (0.06)**	**< 0.001**	**2.68 (1.81)**
	PerLong	**< 0.001**	**1.90 (0.23)**	**< 0.001**	**0.66 (0.05)**	0.053	1.25 (0.11)
	MedGas	**< 0.001**	**1.45 (0.21)**	**< 0.001**	**0.77 (0.04)**	0.273	1.12 (0.13)
SD							
	GlutMed	**< 0.001**	**2.41 (0.47)**	**< 0.001**	**0.69 (0.14)**	**< 0.001**	**1.66 (0.39)**
	TibAnt	**< 0.001**	**6.10 (6.55)**	**< 0.001**	**0.61 (0.05)**	**< 0.001**	**3.72 (3.84)**
	PerLong	**< 0.001**	**2.51 (0.40)**	**< 0.001**	**0.58 (0.04)**	**0.008**	**1.46 (0.25)**
	MedGas	**< 0.001**	**2.00 (0.30)**	**< 0.001**	**0.72 (0.03)**	**< 0.001**	**1.44 (0.22)**

^a^Presented as quotient between the mean values for each dataset (SEM of individual quotients on sample level)

^b^Quotient between VR session 1/control test with eyes open

^c^Quotient between VR session 5/VR session 1

^d^Quotient between VR session 5/control test with eyes open. Statistically significant p-values are shown in bold.

The analysis to determine the effects of adaptation revealed that the older participants significantly decreased from VR session 1 to VR session 5 mean (*p* < 0.001) and SD (*p* < 0.001) normalized EMG amplitudes across the five VR sessions in the four muscles investigated (Fig. 1; Table 3). Mean amplitudes were 23% decreased in GlutMed; 36% decreased in TibAnt; 34% decreased in PerLong and 23% decreased in MedGas. SD EMG amplitudes were 31% decreased in GlutMed; 39% decreased in TibAnt; 42% decreased in PerLong, and 28% decreased in MedGas.

When comparing the mean and SD EMG amplitudes during VR session 5 with the values during the control test with eyes open, mean EMG amplitudes remained significantly higher during VR in two of the investigated muscles (*p* < 0.001) (Figs. 1 and 2; Table 3). Mean amplitudes were 21% higher in GlutMed and 168% higher in TibAnt. SD EMG amplitudes in the four investigated muscles remained significantly higher (*p* ≤ 0.008); 106% higher in GlutMed; 272% higher in TibAnt; 46% higher in PerLong, and 44% higher in MedGas.

### Effects on normalized EMG amplitudes from vision and from age during the control tests

The repeated measures GLM ANOVA analyses revealed that no vision (eyes closed) resulted in a significant increase compared with eyes open in the normalized mean EMG amplitudes in the GlutMed and MedGas muscles (*p* ≤ 0.045) and gave a significant increase in the SD EMG amplitudes in the MedGas muscles (*p* = 0.006) (Fig. 2; Table 4). The mean (*p* < 0.001) and SD (*p* < 0.001) EMG amplitudes were significantly higher in all muscles in the older group than in the young group.

The significant interactions between vision and age group revealed that for no vision the older group had significantly greater mean and SD EMG amplitudes in the TibAnt, muscles compared to the young group (*p* ≤ 0.022) (Table 4).

**Table 4:** Effects on normalized EMG amplitudes from Vision and from age during the control tests

Normalized EMG amplitudes^a^	Mean^b^			SD^b^		
	Vision	Age group	Vision x Age group	Vision	Age group	Vision x Age group
GlutMed	**0.001 [11.6]**	**< 0.001 [100.8]**	0.167 [2.0]	0.059 [3.7]	**< 0.001 [58.8]**	0.061 [3.7]
TibAnt	0.062 [3.6]	**< 0.001 [24.0]**	**0.004 [9.4]**	0.329 [1.0]	**< 0.001 [24.6]**	**0.022 [5.6]**
PerLong	0.343 [0.9]	**< 0.001 [76.0]**	0.657 [0.2]	0.145 [2.2]	**< 0.001 [90.3]**	0.674 [0.2]
MedGas	**0.045 [4.2]**	**< 0.001 [32.0]**	0.331 [1.0]	**0.006 [8.3]**	**< 0.001 [29.4]**	0.178 [1.9]

^a^Repeated measures GLM ANOVA analyses of how the stability was affected by main factor “Vision”, ‘Age group’ and the interaction factor ‘Vision’ x ‘Age group’ during VR

^b^Presented as: *p*-values and [F-values]. The notation “< 0.001” means that the p-value is smaller than 0.001. Found significant effects are marked in bold text

### Effects on EMG amplitudes from vision during the control tests in young adults

Within-subjects analyses revealed that vision did not significantly change normalized mean and SD EMG amplitudes used in the four muscles GlutMed, TibAnt, PerLong and MedGas (Fig. 2; Table 5) in young adults.

**Table 5:** Effects on normalized EMG amplitudes from vision during the control tests in young adults

Normalized EMG amplitude^a^	Mean		SD	
	*p*-value	EC/EO^b^	*p*-value	EC/EO^b^
GlutMed	0.063	1.07 (0.05)	0.745	0.98 (0.05)
TibAnt	0.630	0.92 (0.05)	0.582	1.06 (0.15)
PerLong	0.400	1.03 (0.08)	0.279	1.12 (0.12)
MedGas	0.451	1.02 (0.08)	0.090	1.08 (0.07)

^a^Presented as quotient between the mean values for each dataset (SEM of individual quotients on sample level)

^b^Quotient between the control tests with eyes closed/control tests with eyes open

### Effects on normalized EMG amplitudes from vision during the control tests in older adults

Within-subjects analyses revealed that eyes closed significantly increased normalized mean EMG amplitudes used in three of the four muscles investigated in older adults (*p* ≤ 0.034); 13% increase in GlutMed; 40% increase in TibAnt and 11% increase in MedGas (Fig. 2; Table 6). SD EMG amplitudes in three of the four muscles investigated also increased significantly (*p* ≤ 0.008); 15% increase in GlutMed; 45% increase in TibAnt and 19% increase in MedGas.

**Table 6:** Effects on normalized EMG amplitudes from vision during the control tests in older adults

Normalized EMG amplitude^a^	Mean		SD	
	*p*-value	EC/EO^b^	*p*-value	EC/EO^b^
GlutMed	**0.004**	**1.13 (0.09)**	**0.005**	**1.15 (0.14)**
TibAnt	**0.011**	**1.40 (0.49)**	**0.008**	**1.45 (0.95)**
PerLong	0.096	1.10 (0.05)	0.090	1.05 (0.06)
MedGas	**0.034**	**1.11 (0.07)**	**< 0.001**	**1.19 (0.06)**

^a^Presented as quotient between the mean values for each dataset (SEM of individual quotients on sample level)

^b^Quotient between the control tests with eyes closed/control tests with eyes open. Statistically significant p-values are shown in bold.

## Discussion

The results showed that immersive VR-induced visual stimulation has a pronounced effect on postural muscle activation in the lower extremity, and that the postural control system can adapt with repeated exposure. In the first VR session, both young and older adults exhibited significantly higher EMG amplitudes (mean and variability) in all examined muscles compared to quiet standing with eyes open. This confirms that a conflicting moving visual environment, i.e., in this study a rollercoaster VR stimulus elicits an augmented neuromuscular response to maintain stability. Notably, the older adults, although presented with a less challenging VR rollercoaster movie, showed significantly larger increases, compared to young adults, in mean and SD EMG in all muscles during the initial VR exposure. With repeated VR trials, EMG amplitudes in all muscles declined progressively across sessions 1 through 5 in both age groups. However, adaptation was not absolute. After five trials, mean muscle activity in the VR condition remained elevated above the eyes-open control trial in GlutMed for both groups, and also in TibAnt among the older adults. Interestingly, the variability (SD) of EMG remained significantly higher during VR compared to the eyes-open control trail for all muscles in both groups, even after adaptation, plausibly suggesting that subtle irregularities in muscle recruitment persisted.

### Neuromuscular reaction to VR-induced visual stimulation

Our finding that immersive VR acutely increases postural muscle activation aligns with previous research on postural disturbance. When visual and physical motions are incongruent, the CNS perceives a threat to balance and reflexively augments stabilizing muscle contractions (Acuna et al. 2019; Cano Porras et al. 2021). Prior studies using VR have typically quantified this response in terms of increased sway or torque on a force platform (Fransson et al. 2019; Almqvist Nae et al. 2024). Here, by measuring EMG, we directly captured the neuromuscular strategy underlying that postural response, i.e., greater engagement of ankle and hip muscles to counteract the illusory motion, especially among older adults. Our control test results bolster this interpretation, when standing with eyes closed (removing visual feedback), older adults significantly increased muscle activity in multiple muscles (GlutMed, TibAnt, MedGas), whereas young adults showed no or minimal change. This is in line with previous studies reporting increased EMG activity and co-contraction of ankle muscles among older adults compared to young adults in eyes closed vs. eyes open quiet stance conditions (Benjuya et al. 2004; Baudry and Duchateau 2012). This differential effect confirms that the older group relied more on vision to maintain balance. Removing visual input (or presenting a misleading visual scene in VR) led the older adults to up-regulate muscle activation to avoid losing balance, reflecting a greater concern for stability in the absence of reliable vision compared to young adults, which is discussed in more detail below.

Importantly, the type of visual stimulus used in our study, a dynamic 360° rollercoaster movie, provided continuous, provocative optic flow in multiple directions. This likely engaged a broad range of postural muscles, consistent with our observation that all four recorded muscle groups (hip abductor, ankle plantar- and dorsiflexors, and pronator) showed increased activity. The GlutMed (a hip stabilizer in the mediolateral plane) and PerLong (ankle pronator) are crucial for lateral balance; their activation suggests participants were responding to perceived side-to-side motions in the VR. Meanwhile, increased activity in TibAnt and MedGas (which largely act in antagonistic fashion to control the ankle in the anterior-posterior plane) indicates efforts to counter forward/backward tilts and vertical movements in the visual scene.

The significant increases in the variability (SD) of EMG signal during VR compared to control tests further imply that the muscle responses from the participants were not only increased but also more irregular or intermittent. Increased EMG SD has been reported to correlate positively with force SD in submaximal isometric contractions, indicating increased motor variability and reduced force steadiness (Yuan and Kim 2025).

In practical terms, the initial VR stimuli triggered a state of increased vigilance and muscular activation in both age groups, as they responded to the sensory conflict, in line with impedance control using a stiffening strategy (Franklin and Wolpert 2011). The reduction in EMG amplitudes over the VR-trials indicates a neuromuscular adaptation, and the use of more favorable strategies. The finding that EMG SD was still higher for all muscles in both groups at VR session 5 compared to the control test may indicate a more feedback- or feedforward-based impedance control (Franklin and Wolpert 2011).

### Neuromuscular adaptation to repeated VR-induced visual stimulation

A central novel finding of this study is that repeated exposure to an identical VR stimulation led to a systematic reduction in postural muscle activity, demonstrating a clear acute neuromuscular adaptation. Over five successive trials, participants became progressively less reactive: by the final trial, mean EMG levels had dropped by roughly 16–27% in young adults and 23–36% in older adults relative to the first VR session. Statistically, the main effect of repetition was highly significant for all muscles, and all trended downward. These results are in line with previous research showing decreasing EMG amplitude in postural muscles as an adaptation to repeated or continuous postural perturbations in standing (Patel et al. 2009b; Schmid et al. 2011). Our study extends beyond previous EMG-based studies by using VR and including EMG-measures of muscles controlling both the ankle and the hip, bilaterally. VR enables a feasible and affordable method for standardized postural perturbation. EMG measurements contribute information about postural neuromuscular control and adaptation due to sensory disturbances.

In our study, using repeated visual stimulation, sudden immersive visual motion is given substantial weight by the postural control system (hence the large initial muscle response), but with repeated exposure the CNS learns that this visual input is incongruent with physical reality and reweight sensory input to produce more adapted motor responses. Consequently, CNS down-weights the disruptive visual information and places relatively more trust in the consistent vestibular and somatosensory feedback (which indicates that the body is not truly moving). From a functional perspective, this is reflected in a gradual increase in stability over time, a form of habituation reported in previous balance studies involving repeated visual VR stimuli (Fransson et al. 2019; Almqvist Nae et al. 2024). However, not all VR stimulation seem to instigate adaptive processes (Ahuja et al. 2025). Fransson et al. (2019) observed, using force-platform measures, that healthy young individuals adapt significantly after 4–5 VR exposures, with postural stability energy decreasing by nearly half (Fransson et al. 2019). Our EMG-based findings strongly parallel those results but extend them by revealing how postural muscles are modulated during adaptation and by confirming that these neuromuscular adaptations occur not only in young adults but also in older adults (albeit with some differences). It is encouraging that older participants in our study did exhibit significant adaptation across trials, e.g., the statistical interaction effects indicated that in certain muscles (e.g. PerLong and MedGas) the older group’s EMG decreased more over trials than the young group did. This suggests that older adults had greater room for improvement (since they started at higher activation levels) and were indeed capable of substantially recalibrating their responses. Our results aligns with those of Bugnariu and Fung, indicating that even a single prolonged exposure session can lead to adaptive changes in postural control among older adults, resulting in improved stability despite sensory conflicts (Bugnariu and Fung 2007).

Automatically, repeated VR perturbations likely facilitate a form of implicit balance training where the CNS refines its internal model of the relationship between visual input and self-motion, learns to expect the upcoming perturbations, and adjusts muscle synergies accordingly to avoid overreaction. Consistent with this idea, prior studies have shown that repeated exposure to incongruent visual environments can decrease a person’s visual dependency for balance (Akizuki et al. 2005; Fransson et al. 2019; Almqvist Nae et al. 2024). Akizuki et al. (2005), for example, found that after adaptation in a virtual environment, participants showed decreased sway responses, implying that they had shifted to rely more on somatosensory cues when visual cues became less trustworthy (Akizuki et al. 2005).

### Neuromuscular differences between young and older adults due to VR stimulation

Across all conditions, older adults in our study exhibited higher postural muscle activation than young adults, highlighting distinct age-related control strategies. Notably, this difference existed although the older adults watched a less challenging VR rollercoaster movie, The GLM analysis found a robust main effect of age group: the older group had significantly greater EMG means and variability (SD) in every muscle, whether during quiet stance or VR. This overarching difference is consistent with the well-documented phenomenon that healthy aging is accompanied by increased muscle activation and antagonist co-contraction during balance tasks (Rubega et al. 2021). Greater co-activation can help stabilize joints and compensate for slower reflexes or sensory deficits, effectively “locking” the body to prevent falls (Dos Anjos et al. 2017). However, it also reflects a less efficient motor strategy, as it requires more muscular effort to maintain the same posture and reduce the flexibility to counter balance disturbances. The fact that even in the easiest condition (eyes open quiet standing) the older adults showed higher normalized EMGs than the young adults suggest that they naturally adopt a stiffer, more cautious stance. This likely stems from a combination of factors: age-related loss of sensory acuity (i.e., proprioceptive, vestibular and visual functions), disturbed multisensory integration, reorganization of CNS, decreased muscle strength (requiring a higher fraction of maximum effort to maintain posture), and possibly fear of falling leading to subconscious tensing (Papegaaij et al. 2014; Henry and Baudry 2019; Zhang et al. 2020; Rubega et al. 2021; Wang et al. 2024).

The vision vs. no-vision control tests in our experiment further emphasized the older adults’ dependency on visual input. With eyes closed, older adults had significant increases in EMG (e.g. ~40% increase in TibAnt mean amplitude), indicating that removing vision forced them to engage their muscles more to hold balance. Young adults, in contrast, showed no significant EMG changes with eyes closed, implying that they could rely on non-visual senses effectively. Similar increase in especially TibAnt has been reported among older but not young adults in eyes closed conditions with both wide and narrow stance (Benjuya et al. 2004). These findings resonate with the concept of “visual dependence” increasing with age.

Our data puts a quantitative muscle-based perspective on this: older adults essentially up-regulate neuromuscular drive in the absence of vision as a compensatory mechanism. This age disparity was also evident in the VR stimuli, which can be perceived as a form of distorted vision. The older group’s extreme initial responses (e.g. 520% increase in TibAnt variability during VR session 1 vs. the eyes-open control test) underline that they were far more perturbed by their less challenging rollercoaster VR movie than the young adults by their more challenging rollercoaster VR movie. Interestingly, with adaptation, older adults did show marked reductions in these measures, demonstrating plasticity. Yet even after habituation, their reliance on vision remained apparent, e.g., older adults’ TibAnt activity during VR session 5 was still nearly 170% higher than in normal eyes-open stance, whereas younger adults showed no significant difference.

### Implications for rehabilitation and future research

The present findings carry several implications. First, they validate the use of VR environments to challenge and train balance in different age populations. The fact that participants adapted over multiple VR sessions suggests that repeated practice in an immersive, optically challenging setting can affect postural control. Future research is needed to understand if this translates to daily situations and activities. In a therapeutic context, this supports the idea of using VR as a sensory training tool, e.g., to help older adults or balance-impaired patients learn to cope with visual stimulation and reduce over-reliance on vision.

Our results showed significant improvements, in terms of decreased use of muscle activation, within a single session consisting of five short exposures. Longer-term training across days or weeks might yield even more pronounced gains, potentially translating to better real-world stability (e.g. walking in busy environments or coping with moving crowds). However, future studies are needed to verify the effect of long-term training. The concept is analogous to desensitization therapy: by repeatedly exposing individuals to a sensory conflict (in a safe setting), the CNS learns to resolve the conflict more efficiently. Indeed, prior work has documented that balance training incorporating visual stimulations or odd visual feedback can enhance stability in older adults over time (Almqvist Nae et al. 2024; Wang et al. 2024).

VR provides a flexible and engaging way to deliver such stimuli with precise control over difficulty. One can envisage balance exercise programs where older adults “ride” VR moving scenes or games regularly to exercise their sensory integration faculties. However, care must be taken to tailor the intensity. Our data suggest that older adults react very strongly at first, so a progressive approach (starting with milder visual motions or shorter durations) may be warranted to avoid excessive discomfort or fear that could lead to adverse effects (e.g. falls or refusal to continue). Scientifically, our study contributes novel insights into the muscular-level adaptations that support sensory reweighting processes. Whereas previous research mostly examined center-of-pressure or sway outcomes, this study showed that the brain’s adaptive response involves modulating the output of specific postural muscles. The largest adaptive changes were observed in ankle musculature (e.g. TibAnt and PerLong in older adults dropped by ~ 34–36% in mean EMG over sessions), highlighting the role of ankle strategy in balancing visual stimulations. Meanwhile, hip muscle (GlutMed) also adapted (~ 23% reduction in older adults), suggesting a combined ankle–hip strategy that became more efficient with practice. Interestingly, although the mean level of muscle activation decreased, EMG variability remained elevated during VR stimuli, even after adaptation had occurred. Future research should investigate whether more prolonged or repeated exposures (or perhaps using a predictable vs. random visual stimulation) would eventually normalize EMG variability. Additionally, it would be valuable to measure postural stability or sway alongside EMG in such adaptation paradigms to correlate muscle activation changes with mechanical outcome improvements. While our study focused on EMG amplitude, analyzing muscle latency responses or coordination patterns (synergies) during adaptation could further illuminate how the motor control strategy evolves—for instance, do antagonist muscles become more out-of-phase (reducing co-contraction) with training?

### Limitations

We acknowledge several important limitations in our work. An important limitation of the study was that the younger and older group watched different rollercoaster VR movies. The older participants viewed a less challenging movie compared to the young adults, to reduce potential strain and inability to perform the task. The most likely consequence of this limitation is that the older group responded less to their VR stimulation than they would have if they had watched the same VR movie as the young group. Thus, the presented results are likely an underestimation of the true effects of VR on an older group. However, despite that this study limitation likely increased the threshold of detecting age-related differences, the test conditions used in the study were sufficiently appropriate to reveal numerous significant age-related differences in neuromuscular adaptation and postural muscle activity patterns.

The cohorts in the study, both young and older adults were relatively healthy and highly functioning. Results might differ in frail older persons or those with vestibular, neurological or multisensory deficits, who could have either exaggerated responses or impaired adaptive capacity. All testing was performed in a sequence during one day, and thus, we could not assess retention of adaptation on subsequent days or transfer to other balance tasks, two important aspects if VR were to be used in training regimens. Cybersickness was minimal in our study (no participant dropped out due to VR sickness), likely because the exposures were brief (2 min) and participants could rest as needed between sessions. However, individual variability in tolerance exists; monitoring subjective balance confidence and symptom ratings would be useful in extended VR protocols, especially with older people and participants with specific balance disorders.

In this study we chose to normalize RMS EMG amplitude to MVC. This is an established and commonly suggested method. However, there should be some caution in the interpretation since the normalized values are influenced by the ability and willingness to perform the true maximum contractions during the MVC tests, which may have differed between the groups (Besomi et al. 2020). Initially, we considered normalizing the EMG from the eyes closed control and VR tests to the control test with eyes open. That method would, however, not have supplied any information about the EMG amplitude during the tests in relation to maximal contraction, which we considered relevant in this study.

Finally, we assessed EMG amplitude of postural muscles of the ankles (MedGas, TibAnt and PerLong), and the hips (GlutMed). Although these muscles are considered highly important for postural control in standing, further details on the neuromuscular adaptations during VR stimulation could be explored by measuring EMG on core trunk musculature.

## Conclusions

Our findings showed that visual VR stimulation significantly increases postural muscle activity compared to normal quiet stance, and that the postural control system exhibits neuromuscular adaptation (decreased muscle activation) with repeated exposures to the same VR stimulus. This was shown in both young and older adults, although older adults displayed larger normalized EMG values and a less complete adaptation over the five VR stimulations. The main novelty of this study lies in demonstrating the neuromuscular adjustments, in terms of EMG amplitudes of key stabilizing muscles, that underlie the habituation to immersive visual stimulations. These findings deepen our understanding of how the CNS resolves sensory conflicts over time and highlights age-related differences in sensorimotor integration. Taken together, this supports the use of VR in balance training, e.g., among older adults to improve physical functioning and reduce the risk of falling. Future research should explore longer-term training effects, optimal exposure regimes, and whether adaptation in the virtual environment translates to improved balance in real-world scenarios. VR-based interventions could potentially reduce maladaptive visual dependence and enhance overall postural stability in populations at risk of falls. The present study demonstrates that while immersive visual input poses a challenge to the neuromuscular system, it is also capable of adapting through repeated exposure.

## Data Availability

Research data will be shared upon request to the authors.
